# NIS-Centered Reporter Gene Imaging and Radionuclide-Integrated Nanoplatforms for Quantitative Tracking of Immune Cell Therapy in Oncology and Inflammatory Disease Models

**DOI:** 10.3390/ph19050790

**Published:** 2026-05-18

**Authors:** Sang Bong Lee

**Affiliations:** 1 SimVista Inc., Cheongju-si 28161, Republic of Korea; sblee@simvista.co.kr or sang2949@dongguk.edu; 2 Department of Biomedical Sciences, Medical School, Chonnam National University, Hwasun 58128, Republic of Korea; 3 BioMedical Sciences Graduate Program (BMSGP), Chonnam National University, Hwasun 58128, Republic of Korea; 4 Department of Life Science, Dongguk University-Seoul, Goyang-si 10326, Republic of Korea

**Keywords:** NIS reporter gene, immune cell tracking, positron emission tomography/single-photon emission computed tomography imaging, radionuclide-integrated gold nanoparticles, radiotheranostics, Cerenkov luminescence imaging, tumor-on-a-chip, adoptive cell therapy, CAR-T cells, CAR-NK cells

## Abstract

Cell-based immunotherapies require noninvasive tools that can quantify the migration, biodistribution, and persistence of administered immune cells. This review focuses primarily on oncologic immune cell therapy, while also considering selected inflammatory disease models in which immune-cell trafficking is biologically relevant. We critically compare direct radionuclide labeling, sodium iodide symporter (NIS)-based reporter gene imaging, radionuclide-integrated nanoplatforms, and Cerenkov-based hybrid optical conversion strategies. Direct labeling with agents such as [^89^Zr]Zr-oxine, [^111^In]In-oxine, and [^99^ᵐTc]Tc-HMPAO enables early positron emission tomography (PET)/single-photon emission computed tomography (SPECT) biodistribution assessment, usually within hours to several days after cell administration. NIS reporter imaging with [^124^I]NaI, [^123^I]NaI, [^99^ᵐTc]TcO_4_^−^, or [^18^F]TFB supports repeated viability-dependent imaging, because signal generation depends on active transporter expression in living engineered cells. Radionuclide-integrated gold nanoplatforms can improve intracellular retention and offer theranostic potential through combined imaging, photothermal, radiotherapeutic, or immunomodulatory functions. We further discuss PET/SPECT balance, radiopharmaceutical nomenclature, nanoparticle stabilization, ethical aspects of genetic modification, tumor-on-a-chip systems for preclinical testing, and limitations of narrative evidence synthesis. Together, these platforms provide complementary strategies for image-guided immune cell therapy, with translational relevance for patient selection, treatment optimization, safety monitoring, and oncology practice. In conclusion, NIS-centered nuclear imaging and radionuclide-integrated nanoplatforms represent complementary, clinically actionable tools for quantitative immune-cell tracking, therapeutic optimization, and safety monitoring in translational oncology and inflammatory disease research.

## 1. Introduction

Cell-based immunotherapy has become a major therapeutic modality in cancer and inflammatory diseases through the use of engineered or expanded immune cells that engage and eliminate pathological targets [1,2,3]. Examples include adoptive transfer of T lymphocytes, natural killer (NK) cells, and dendritic cell vaccines, which have demonstrated promising efficacy in clinical trials and expanded treatment options for patients with refractory malignancies [4,5,6,7]. Despite these advancements, the lack of reliable, noninvasive methods to monitor the in vivo fate of administered immune cells has limited broader clinical application and effective optimization of treatment strategies [8,9,10,11,12].

Accurate tracking of therapeutic cells after infusion is essential for understanding mechanisms of action, assessing therapeutic engagement at target tissues, confirming biodistribution, and evaluating persistence over time [13,14,15]. Traditional clinical assessments such as biopsies and peripheral blood analyses provide only static or partial information at a single time point, without capturing whole-body dynamics of cell trafficking and functional behavior [16,17,18]. To address these limitations, imaging methodologies that enable longitudinal, quantitative, and whole-body visualization have become critically important in preclinical and clinical research.

Despite rapid progress in immune cell imaging, existing reviews often discuss direct radiolabeling, reporter gene imaging, radionuclide-integrated nanomaterials, and optical conversion strategies as separate methodological areas [19,20,21,22]. The novelty of this review is its NIS-centered and translationally oriented integration of these approaches for immune cell therapy tracking, with explicit comparison of PET and SPECT options, viability-dependent reporter readouts, chelator-free radionuclide-integrated nanoplatforms, and hybrid optical conversion.

Among current imaging modalities, nuclear molecular imaging—comprising positron emission tomography (PET) and single-photon emission computed tomography (SPECT)—is particularly suited for cell tracking because it combines high sensitivity, deep tissue penetration, and quantitative whole-body assessment [19,20,21,22]. PET offers higher spatial resolution and quantitative accuracy for tracers such as [89Zr]Zr-oxine, [124I]NaI, and [18F]TFB, whereas SPECT remains clinically important because of established leukocyte-labeling protocols, broad gamma-camera availability, and compatibility with [111In]In-oxine, [99ᵐTc]Tc-HMPAO, [123I]NaI, and [99ᵐTc]TcO4 [23,24]. Therefore, the revised manuscript treats PET and SPECT as complementary rather than competing modalities.

Early approaches for tracking therapeutic cells involved direct ex vivo labeling with radiotracers prior to infusion. Traditional methods have utilized radionuclides such as [111In]In, [89Zr]Zr, and [64Cu]Cu linked to chelators or nanoparticles, enabling initial biodistribution and trafficking evaluation after adoptive transfer [25,26,27,28,29,30]. These direct radiolabeling methods have provided valuable insights into cell distribution and homing patterns in vivo; however, limitations such as radiolabel efflux from cells, signal dilution with cell division, and restriction by radionuclide half-life have constrained long-term monitoring capability [31,32,33].

To overcome these limitations, nanotechnology-based radiolabeling strategies have been developed to improve intracellular retention and imaging stability. Among them, radionuclide-embedded gold nanoparticles (RIe-AuNPs) have shown enhanced radiochemical stability, high radiolabeling efficiency, and minimal perturbation to cell viability and function [34,35,36,37,38]. These nanoplatforms permit sensitive PET and Cerenkov luminescence imaging of labeled immune cells, including dendritic cells, macrophages, NK cells, and even platelets in preclinical models, while preserving key biological properties necessary for effective immunotherapy [39,40,41,42]. Furthermore, advanced surface chemistry and core design have enabled long-term retention of radionuclides inside labeled cells, addressing a major challenge associated with conventional direct labeling approaches [43,44,45].

In parallel with direct labeling, genetic reporter systems have been developed to facilitate in vivo tracking beyond the physical decay constraints of direct radiolabels. Reporter genes stably introduced into therapeutic cells allow accumulation of radiotracers through cellular expression mechanisms, supporting repeated imaging at multiple time points after administration [46,47,48,49]. Among available reporters, the sodium iodide symporter (NIS) has been one of the most widely studied for nuclear imaging applications due to its ability to mediate active uptake of imaging tracers such as radioiodine and [18F]tetrafluoroborate (TFB) [50,51,52,53]. NIS-based reporter gene imaging enables quantitative visualization of cell migration to lymph nodes, tumor microenvironments, and other sites of therapeutic relevance in vivo, and has been successfully applied across a range of immune cell types [54,55,56].

Critical aspects in selecting between direct labeling and reporter gene strategies include the desired imaging time frame, potential effects on cell phenotype, and technical feasibility for clinical translation [57,58]. Direct labeling offers simplicity and immediate implementation, whereas reporter gene techniques promise longitudinal monitoring with reduced signal loss and the ability to perform repeat imaging over extended periods. Both approaches continue to evolve with advances in radiochemistry, tracer development, and cellular engineering.

**Figure 1:**
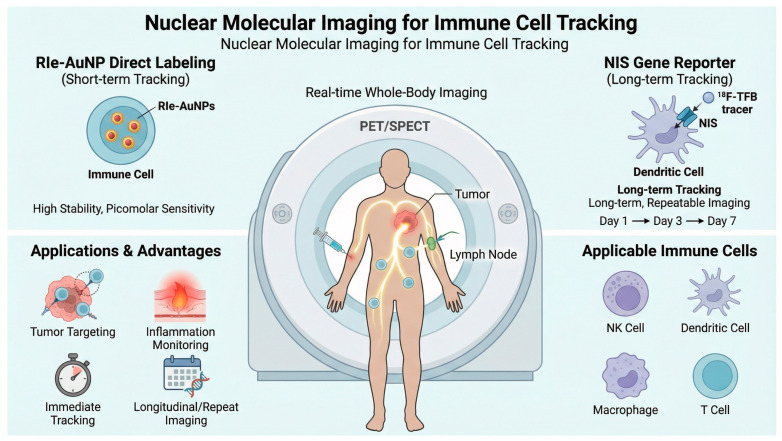
Nuclear molecular imaging strategies for tracking therapeutic immune cells in vivo.

In this review, we summarize translational research efforts focused on the integration of chelator-free radionuclide-embedded nanoplatforms and NIS-based reporter gene systems for immune cell tracking. We highlight design principles for achieving stable radiolabeling, methods that preserve cell viability and immune function, and imaging applications that provide quantitative insight into immune cell behavior in vivo. Finally, practical considerations and future directions in the application of nuclear molecular imaging to immune cell-based therapies are discussed (Figure 1).

Figure 1 provides a schematic overview of complementary nuclear imaging approaches for monitoring adoptively transferred immune cells. Direct ex vivo labeling with radionuclide-integrated gold nanoparticles (RIe-AuNPs) supports short-term positron emission tomography/single-photon emission computed tomography (PET/SPECT) assessment of biodistribution [19,20,21,22,23,24,35,36,37,38,39,40,41,42,43,44,45,46,47,48,49,50,59,60,61,62,63,64,65,66]. Sodium iodide symporter (NIS) reporter gene imaging enables repeated tracer-based visualization of viable engineered cells using PET or SPECT tracers [1,2,3,4,5,6,7,8,9,10,11,12,13,14,15,16,17,18,25,26,27,28,67,68,69,70,71,72,73,74,75,76,77,78,79,80,81,82,83]. The graphical presentation is organized into three functional components: (i) RIe-AuNP-based direct labeling for short-term tracking, (ii) PET/SPECT scanner-based whole-body biodistribution analysis, and (iii) NIS reporter-based long-term viable-cell imaging, with representative immune-cell types and translational applications shown in the side panels. Arrows indicate cell administration, trafficking, and signal-generation pathways; isotope symbols indicate radionuclide labeling or tracer uptake; and PET, SPECT, computed tomography (CT), Cerenkov luminescence imaging (CLI), NIS, and RIe-AuNPs are defined at first use in this caption and listed in the Abbreviations list.

## 2. Clinical Rationale and Translational Need for Immune Cell Tracking

Cell-based immunotherapies, including adoptive T-cell transfer, chimeric antigen receptor (CAR) T-cell therapy, natural killer (NK) cell therapy, dendritic-cell vaccines, and stem cell-based delivery systems, have expanded treatment options for cancer and immune-mediated disorders. However, clinical responses remain variable. Therapeutic efficacy depends not only on the cytotoxic or immunomodulatory activity of the administered cells but also on their in vivo distribution, target-site accumulation, persistence, expansion, and off-target localization.

Current monitoring methods provide incomplete information. Tumor biopsy is invasive, regionally restricted, and unsuitable for repeated whole-body assessment. Peripheral blood analysis is less invasive but does not reliably reflect cell behavior within tumors, lymphoid tissues, or inflamed organs. These limitations support the need for imaging methods that can noninvasively measure cellular kinetics throughout the body over time.

Nuclear imaging techniques offer several advantages in this context, including high detection sensitivity, unrestricted tissue penetration, and quantitative signal measurement. Within this framework, the sodium iodide symporter (NIS) has emerged as a particularly well-characterized and clinically grounded reporter system. Initially identified and cloned as the thyroid iodide transporter in 1996 [1,2], NIS was subsequently shown to mediate active sodium-dependent iodide transport with defined stoichiometry [3]. Detailed biochemical and physiological studies established its functional role in thyroid follicular epithelium and lactating mammary tissue [4,5,6,7], while further investigations clarified regulatory mechanisms and broader medical implications [8,9].

The longstanding clinical use of radioiodine for diagnostic imaging and therapy has provided extensive experience with NIS-mediated radionuclide uptake, conferring a degree of translational maturity uncommon among molecular reporter systems. Moreover, NIS expression has been detected in extrathyroidal tissues and a range of malignancies, including breast carcinoma [10,11], demonstrating that its functional activity is not restricted to endocrine physiology. This adaptability supports its application as a genetically introduced reporter in diverse cellular contexts.

The transformation of NIS from an endogenous transporter to a molecular imaging reporter began with the concept of “radioisotope concentrator gene therapy,” which demonstrated that enforced NIS expression enables non-thyroidal tumor cells to accumulate radionuclides [12]. Subsequent studies employing adenoviral vectors confirmed functional expression across multiple tumor models [13,14]. A critical advance was the demonstration that human NIS (hNIS) could serve as a positron emission tomography (PET) reporter gene, permitting noninvasive visualization and quantification of transgene expression in vivo [15]. Follow-up investigations using I-124 PET/CT further established the feasibility of whole-body imaging and quantitative assessment of NIS-expressing tissues in preclinical settings [16,17,18,67,68], thereby laying the foundation for longitudinal reporter imaging.

The introduction of [18F]tetrafluoroborate ([18F]TFB) significantly expanded the clinical applicability of NIS-based imaging. As a PET tracer targeting NIS, [18F]TFB provides high spatial resolution and favorable pharmacokinetics compatible with routine clinical PET workflows [69]. Human studies have characterized its biodistribution and radiation dosimetry [70,71], and both experimental and clinical investigations have demonstrated its ability to detect NIS-expressing tumors with improved diagnostic performance relative to conventional iodine scintigraphy [25,72]. The availability of a clinically validated fluorine-18–labeled tracer strengthens the feasibility of translating NIS-based imaging strategies to immune cell tracking.

**Figure 2:**
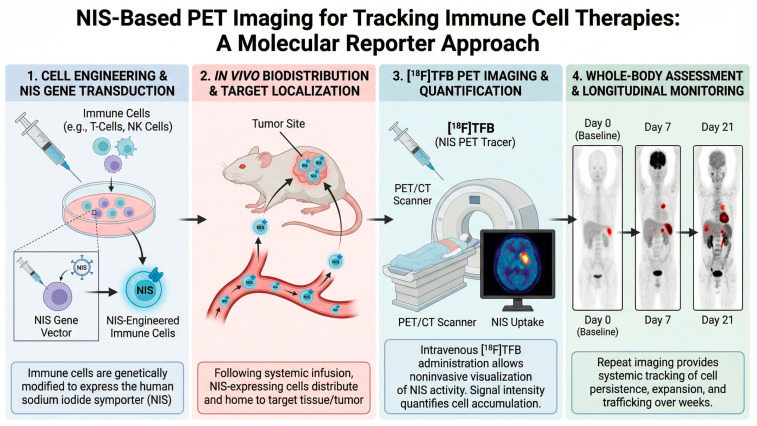
Sodium iodide symporter (NIS)-based positron emission tomography (PET) imaging for longitudinal tracking of engineered immune cells.

Taken together, three interrelated components underpin the rationale for employing nuclear imaging in immune cell-based therapies: a thoroughly defined membrane transporter with extensive clinical precedent [1,2,3,4,5,6,7,8,9], experimental validation of its utility as a quantitative reporter gene [12,13,14,15,16,17,18,67,68], and the development of PET tracers supported by human safety and dosimetry data [25,69,70,71,72]. Compared with localized tissue sampling or surrogate circulating markers, PET/SPECT enables systemic, repeatable evaluation of cellular distribution and persistence. For these reasons, nuclear molecular imaging represents a rational and increasingly integral tool for advancing the development and refinement of immune cell therapeutics (Figure 2, Table 1).

## 3. Radionuclide-Based Immune Cell Tracking: From Direct Labeling to Reporter Gene and Hybrid Optical Conversion Strategies

Radionuclide-based immune cell tracking has progressed through distinct technological phases, beginning with direct ex vivo radiolabeling, advancing to reporter gene imaging exemplified by the sodium iodide symporter (NIS), and expanding toward nanomaterial-integrated and Cerenkov-based hybrid imaging approaches. These developments have shaped the molecular and translational basis for longitudinal in vivo monitoring of adoptively transferred immune cells.

Direct radiolabeling was the first clinically feasible strategy for quantitative immune cell tracking. Chelator-based intracellular labeling systems, particularly [89Zr]Zr-oxine, enabled positron-emitting radionuclides to be incorporated into viable immune cells for PET imaging [43,44,45]. Kit-based good manufacturing practice (GMP) production improved reproducibility and supported clinical translation [46,50]. This approach has been applied to CAR-T cells [44], Vγ9Vδ2 T cells [47], and NK or CAR-NK cells [49], and has helped define in vivo detection thresholds [48]. Its main advantage is procedural simplicity without genetic manipulation. However, radiolabel efflux, signal dilution during proliferation, physical decay, and potential radiotoxicity restrict long-term monitoring. Direct labeling therefore remains most useful for short-term trafficking and early biodistribution assessment.

SPECT-based strategies remain important for immune cell tracking because of their clinical availability, established gamma-camera infrastructure, and compatibility with several direct-labeling and reporter-gene tracers. Autologous leukocyte imaging with [111In]In-oxine and [99ᵐTc]Tc-HMPAO has long been supported by procedural guidelines that describe cell isolation, radiolabeling, quality control, sterility, reinjection, and image acquisition [23,24]. Although these methods were developed primarily for infection and inflammation imaging, they provide an important translational precedent for ex vivo immune-cell labeling, biodistribution assessment, and safety evaluation. For NIS reporter imaging, SPECT-compatible substrates such as [123I]NaI and [99ᵐTc]TcO4− complement PET substrates such as [124I]NaI and [18F]TFB, allowing investigators to select tracers according to availability, half-life, radiation burden, scanner platform, and clinical workflow [23,24,59,73,74,75,76,77,78,79].

Reporter gene imaging was developed to address these limitations. Among available systems, NIS has demonstrated the most advanced degree of clinical translation. Following its cloning and molecular characterization in 1996 [1,2] and subsequent clarification of transport stoichiometry and substrate specificity [3], the biological foundation for iodide-based imaging was established. Further investigations defined its physiological regulation and medical relevance [4,5,6,7,8,9]. The introduction of the “radioisotope concentrator gene” concept [12,13,14] demonstrated that enforced NIS expression permits radionuclide accumulation in non-thyroidal tissues. Validation of human NIS (hNIS) as a PET reporter gene using [124I]NaI enabled noninvasive visualization and quantification of transgene expression in vivo [15,16,17]. Additional studies confirmed quantitative PET imaging capability [68] and extended applications to cardiac cell transplantation models [18,67]. Unlike direct labeling, NIS-mediated imaging generates signal only in viable cells and is not subject to dilution during proliferation, supporting repeated longitudinal assessment.

The development of [18F]tetrafluoroborate ([18F]TFB) further strengthened clinical applicability by providing a PET-compatible NIS tracer with favorable imaging characteristics and human dosimetry data [69,70,71]. SPECT-compatible NIS substrates, including [123I]NaI, [131I]NaI, and [99ᵐTc]TcO4−, also remain relevant for centers without PET access or for specific longitudinal protocols. Clinical investigations have demonstrated improved diagnostic performance of [18F]TFB PET/CT relative to conventional [131I]iodine scintigraphy in selected thyroid cancer settings [25]. Beyond imaging, NIS expression enables therapeutic radioiodine uptake, creating opportunities for integrated imaging and radiotherapeutic strategies [26,27].

In parallel with gene-based strategies, radionuclide-embedded nanomaterials have been explored as alternative immune cell imaging platforms. Gold nanoparticle systems labeled with [99ᵐTc]Tc, [198Au]Au, [64Cu]Cu, [89Zr]Zr, or radioiodine demonstrate chelator-independent stability and theranostic potential [35,36,37,38,39,40,41,42]. These platforms have been applied to visualize dendritic cell trafficking and immune cell maturation [39,40], offering enhanced signal retention compared with conventional chelator-based methods. Long-term intracellular stability and safety profiles, however, require continued investigation.

In this review, the term theranostic refers to a single platform or paired strategy that combines diagnostic imaging with therapeutic capability. For radionuclide-integrated nanoplatforms, this may include simultaneous immune cell tracking and radiation-mediated or immune-modulating effects. For NIS-based systems, it may include reporter imaging combined with therapeutic radioiodine delivery or controlled elimination of engineered cells when clinically necessary.

Cerenkov luminescence imaging (CLI) represents a further conceptual development by converting radioactive decay energy from positron-emitting isotopes into detectable optical photons [51,52,53,54]. This approach enables sensitive optical detection in small-animal models and provides a cost-efficient complement to conventional nuclear imaging. Advances in tomographic reconstruction [55], nanoparticle-mediated modulation [57], and radiochemistry applications [58] have broadened its experimental utility. Limited optical tissue penetration currently restricts deep-tissue clinical applications, but CLI offers a hybrid interface between nuclear and optical imaging modalities.

**Figure 3:**
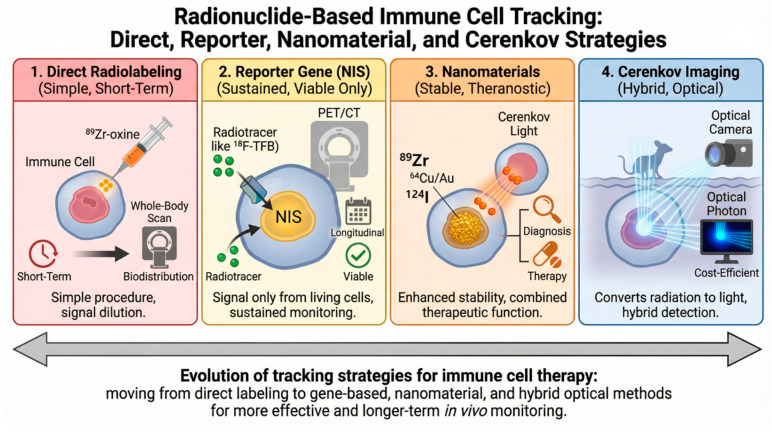
Conceptual evolution of radionuclide-based immune cell tracking strategies.

Radionuclide-based immune cell tracking has therefore evolved from short-term biodistribution mapping toward biologically sustained reporter gene imaging and multifunctional theranostic platforms. Direct radiolabeling established feasibility and quantitative parameters; NIS-based systems enabled viability-dependent longitudinal monitoring; nanomaterial approaches introduced structural stability and integration of therapeutic functions; and Cerenkov methodologies expanded detection strategies. These advances define the present framework for imaging-guided development of immune cell therapeutics (Figure 3, Table 2).

## 4. Comparative Functional Roles of Radionuclide-Based Immune Cell Imaging Platforms

Radionuclide-based approaches for immune cell tracking are best interpreted according to their biological readout and translational applicability rather than as successive technological iterations. Each platform provides distinct information regarding cell distribution, viability, or functional persistence, and their utility varies according to experimental objective and clinical stage.

### 4.1. Direct Radiolabeling and Early Biodistribution Assessment

Ex vivo radiolabeling with [89Zr]Zr-oxine [43,44,45,59] established the practical basis for quantitative PET-based cell tracking in vivo and has been applied to CAR-T cells, γδ T cells, CD8+ T cells, NK/CAR-NK cells, plasma cells, and monocyte-macrophage lineage cells [60,61,62,63,64]. SPECT labeling with [111In]In-oxine and [99ᵐTc]Tc-HMPAO provides an established clinical comparator and remains relevant when PET infrastructure or PET tracer production is unavailable [23,24]. These studies collectively define practical detection limits, short-term trafficking windows, radiotoxicity concerns, and workflow requirements for adoptive cell therapy imaging.

In current practice, direct labeling is primarily applied to immediate post-infusion biodistribution analysis, dose-escalation evaluation, and assessment of unintended organ accumulation during early-phase trials. Because imaging signal reflects the initial intracellular radionuclide load rather than active biological processes, the technique captures distribution kinetics within a limited temporal window. Signal attenuation occurs through isotope decay, efflux, and dilution during cell proliferation. Accordingly, direct radiolabeling is most informative for short-term pharmacokinetic profiling rather than for longitudinal evaluation of cell persistence.

### 4.2. Reporter Gene Imaging and Viability-Dependent Signal Generation

Reporter gene strategies were introduced to overcome the temporal constraints of ex vivo labeling. NIS-based imaging represents the most extensively validated radionuclide reporter system in this category because it has a human origin, lacks enzymatic tracer metabolism, and can be interrogated by both PET and SPECT tracers [74,75,76,77,78,79]. Following its cloning [1,2] and characterization of sodium-dependent iodide transport [3], detailed analyses of regulation and physiological expression patterns established a mechanistic foundation for translational application [4,5,6,7,8,9,84,85]. Functional expression in extrathyroidal tissues [10,11,86,87] further supported adaptability across diverse cellular contexts.

Experimental demonstration that ectopic NIS expression mediates radionuclide accumulation in non-thyroidal tissues [12,13,14] was followed by PET-based validation of human NIS as a quantitative reporter [15,16,17,18,67,68]. In contrast to direct labeling, tracer uptake depends on active transporter expression in viable cells, enabling repeated imaging independent of proliferation-associated signal dilution.

The introduction of [18F]tetrafluoroborate [69,77,78] provided a PET-compatible tracer with favorable imaging characteristics. Human biodistribution and dosimetry studies confirmed safety [70,71,78], and comparative analyses demonstrated improved lesion detection relative to conventional [131I]NaI scintigraphy [25]. Recent NIS-based CAR-NK and regulatory T-cell studies further illustrate the value of reporter gene imaging for longitudinal assessment of viable therapeutic cells in oncology and immune-regulation models [79,80]. NIS expression additionally permits therapeutic radioiodine uptake [26,27], while regulation by oncogenic signaling pathways such as BRAFV600E [28] highlights biologically relevant modulation. Incorporation of NIS into viral vectors and oncolytic systems [29,30,31,32,33,34,81,82,83] has enabled noninvasive monitoring of gene delivery and radio-virotherapy. Collectively, these data support NIS reporter imaging as a clinically adaptable platform for longitudinal and viability-dependent cell tracking.

### 4.3. Radionuclide-Integrated Nanomaterials and Intracellular Signal Retention

Radionuclide-embedded nanomaterials address signal instability through structural incorporation of radioisotopes. Gold-based nanoparticles labeled with [99ᵐTc]Tc, [198Au]Au, [64Cu]Cu, [89Zr]Zr, or radioiodine [35,36,37,38,39,40,41,42] demonstrate chelator-independent retention, reducing intracellular efflux relative to conventional radiolabeling techniques.

Nanoparticle stabilization depends on both radionuclide incorporation chemistry and particle physicochemical design. In chelator-free gold nanoplatforms, the radionuclide can be embedded within, adsorbed onto, or trapped by the metallic shell or core–shell interface, thereby reducing detachment from the carrier compared with conventional surface chelation [39,40,41,42,65,66]. Surface ligands such as tannic acid, polyethylene glycol, DNA or peptide-based coatings, albumin, and amphiphilic polymers can modulate colloidal stability, serum-protein adsorption, cellular uptake, intracellular trafficking, and immune-cell compatibility. For immune-cell tracking, stabilization must therefore be evaluated using radiochemical stability, hydrodynamic size, zeta potential, serum challenge, intracellular retention, radiometabolite release, cell viability, phenotype preservation, cytokine production, antigen-presenting function, cytotoxicity, and in vivo clearance. These parameters are essential because a stable extracellular radiolabel does not necessarily guarantee intracellular retention or preservation of therapeutic immune-cell function after adoptive transfer.

These systems eliminate the need for genetic modification and aim to prolong intracellular radionuclide retention. However, long-term biocompatibility, degradation pathways, and regulatory assessment remain areas requiring further investigation. At present, radionuclide-integrated nanomaterials are best considered signal-retention enhancers with potential theranostic applications.

### 4.4. Cerenkov Luminescence Imaging and Optical Signal Conversion

Cerenkov luminescence imaging (CLI) detects optical photons generated by β−-particle decay [51,52]. Quantitative methodologies have correlated optical intensity with tracer concentration and absorbed radiation dose [53,54]. Technical advances in tomographic reconstruction [55], theoretical modeling [56], nanoparticle-assisted spectral modulation [57], and radiochemistry applications [58] have expanded its preclinical utility.

**Figure 4:**
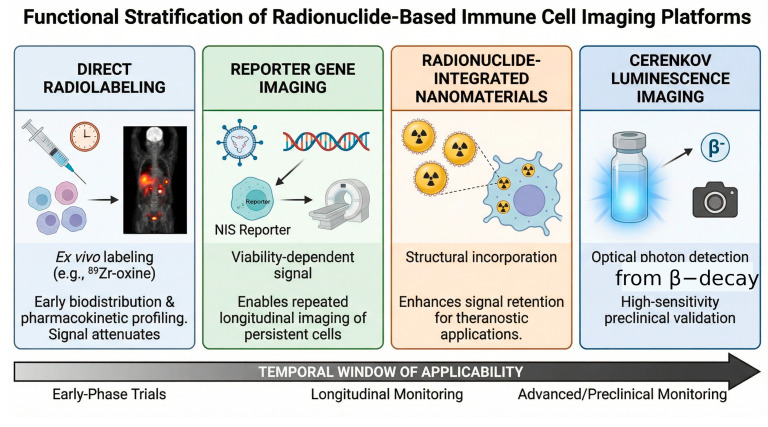
Functional stratification of radionuclide-based immune cell imaging platforms. Direct radiolabeling measures early biodistribution, NIS reporter imaging measures viable-cell persistence, radionuclide-integrated nanomaterials enhance intracellular signal retention, and CLI provides an optical surrogate of radionuclide distribution in preclinical models. The lower panel summarizes the approximate temporal window of each platform. Symbols and abbreviations are defined in the figure legend and Abbreviations list.

Limited tissue penetration restricts CLI in deep human imaging; however, the method provides high-sensitivity detection in small-animal studies and facilitates cross-validation with PET-based measurements. CLI therefore serves as an adjunct modality for experimental and hybrid imaging applications (Figure 4, Table 3).

Hybrid optical conversion strategies exploit photons produced when charged particles emitted by radionuclides travel faster than the phase velocity of light in biological media. In preclinical systems, CLI can be paired with PET or SPECT to validate radiotracer distribution, evaluate cell-labeling stability, or screen nanomaterials that shift, amplify, or quench the optical signal [51,52,53,54,55,56,57,58]. Cerenkov radiation can also excite secondary fluorophores or nanoparticle transducers, creating a bridge between radioactive decay, optical readout, and phototherapeutic activation. However, tissue absorption and scattering restrict deep-tissue human use, so CLI should be interpreted mainly as a mechanistic and translational research tool rather than a replacement for tomographic nuclear imaging.

### 4.5. Modality Selection in Immune Cell Imaging

Selection of an imaging strategy should be guided by the biological endpoint under investigation and the clinical phase of development. Direct radiolabeling is appropriate for early biodistribution and safety assessment. Reporter gene imaging enables repeated evaluation of viable cell persistence. Nanomaterial-based systems may enhance signal stability or support theranostic design. Cerenkov-based methods are primarily applicable to preclinical validation and multimodal integration. Considered collectively, these approaches provide complementary tools for quantitative investigation of immune cell kinetics in vivo.

## 5. Current Diagnostic and Technical Barriers

Although radionuclide-based immune cell imaging is highly promising, several barriers currently restrict broad clinical use. Direct radiolabeling is limited by radiolabel efflux, isotope decay, signal dilution during cell proliferation, and potential radiotoxicity at high labeling activities. Reporter gene imaging addresses some of these issues but introduces different challenges, including vector safety, expression stability, immune recognition of engineered cells, and regulatory review of genetically modified cell products. NIS imaging also requires careful interpretation because physiological tracer uptake occurs in the thyroid, salivary glands, stomach, and urinary tract.

Quantitative standardization remains another major challenge. Imaging signals can vary according to scanner calibration, reconstruction method, injected activity, timing after tracer administration, cell type, labeling efficiency, and background organ uptake. Cross-study comparison is therefore difficult unless acquisition protocols, dosimetry calculations, and reporting metrics are harmonized. Clinically, workflow complexity, radiation exposure, GMP manufacturing, and tracer availability must also be considered before these methods can be routinely incorporated into immune cell therapy trials.

Ethical and regulatory aspects of reporter gene imaging should be explicitly considered. Genetic introduction of NIS or other reporter genes into therapeutic immune cells may raise concerns related to vector selection, insertional mutagenesis, immunogenicity, off-target expression, persistence of modified cells, incidental detection of ectopic uptake, patient consent, and long-term follow-up [22,82,83,88,89,90]. These issues are particularly relevant for CAR-T and CAR-NK products, in which the reporter construct may be integrated into an already genetically modified therapeutic cell. Future clinical translation should therefore include validated vector design, release testing, replication-competent virus testing when applicable, predefined stopping rules, radiation dosimetry, privacy protection for serial imaging data, and transparent communication of imaging-related risks and benefits to patients.

## 6. Future Perspectives

The integration of nuclear molecular imaging with nanotechnology and genetic engineering has expanded the methodological framework for monitoring immune cell-based therapies. Although direct radiolabeling and reporter gene approaches have demonstrated feasibility in both preclinical and early clinical settings, several areas require further investigation to facilitate broader clinical translation.

Microphysiological systems and 3D tumor models represent an important emerging interface between radiopharmaceutical development and immune cell therapy. Conventional two-dimensional cultures often fail to reproduce tumor-cell density, extracellular matrix organization, oxygen and nutrient gradients, interstitial pressure, stromal interactions, vascular perfusion, and immune-cell infiltration. Three-dimensional spheroids, organoids, bioprinted tumor constructs, and tumor-on-a-chip platforms can more closely approximate these features and may help evaluate radiotracer penetration, receptor accessibility, radionuclide retention, absorbed-dose heterogeneity, immune-cell trafficking, and treatment response before animal experiments [91,92,93,94,95,96,97]. For radiotheranostic drug development, microfluidic tumor-on-a-chip systems are particularly attractive because they can model dynamic pharmacokinetic exposure, spatial gradients, repeated sampling, and co-culture of tumor, endothelial, stromal, and immune compartments. Integration of PET/SPECT-compatible radiotracers, Cerenkov readouts, autoradiography, and digital dosimetry with these platforms may reduce the gap between conventional in vitro screening and in vivo validation.

First, quantitative imaging strategies incorporating dosimetric and kinetic modeling warrant continued development. As adoptive cell therapies are increasingly evaluated in combination regimens and earlier disease stages, imaging methodologies should extend beyond qualitative biodistribution assessment toward quantitative characterization of cell trafficking, expansion, and persistence. The application of compartmental analysis, voxel-based dosimetry, and serial PET measurements may allow correlation between imaging-derived parameters and therapeutic response or toxicity. Such approaches may contribute to optimized cell dosing and treatment scheduling.

Second, refinement of radionuclide selection and tracer design remains an important objective. Radionuclides with half-lives compatible with immune cell kinetics, favorable positron emission characteristics, and acceptable radiation burden are necessary for routine clinical application. In NIS-based systems, continued optimization of [^18^F]F-labeled tracers and evaluation of alternative PET isotopes may improve image contrast and reduce physiologic background activity. Advances in radiochemistry, including site-specific labeling and chelator-free strategies, may further enhance intracellular retention and signal stability in nanoparticle-based platforms.

Third, theranostic integration represents a rational extension of current imaging strategies. Radionuclide-embedded nanomaterials have demonstrated potential not only for cell tracking but also for modulation of immune cell function, including effects on dendritic cell maturation and macrophage polarization. In parallel, NIS expression enables targeted radioiodine delivery, providing a potential mechanism for selective elimination of engineered cells in the context of adverse events. The combination of imaging capability with controlled therapeutic intervention may improve both safety and mechanistic understanding of immune cell therapies.

Fourth, multimodal imaging approaches may support translational development. Hybrid strategies incorporating PET with optical or other imaging modalities can provide complementary information in preclinical models. Although optical techniques such as Cerenkov luminescence imaging are limited by tissue penetration in humans, they remain valuable for mechanistic studies, tracer validation, and radiochemistry development. Further exploration of multimodal reporter systems may enhance spatial and functional characterization of therapeutic cells.

Fifth, standardization of manufacturing, imaging protocols, and regulatory pathways is essential for clinical implementation. The availability of GMP-compliant radiolabeling kits, validated gene transfer methods, and harmonized quantitative imaging criteria will be critical for reproducibility across institutions. Reporter gene-based approaches in particular require rigorous evaluation of vector safety, expression stability, and long-term monitoring considerations. The established clinical use of NIS-targeting tracers and radioiodine provides a practical foundation for regulatory acceptance.

Overall, future progress will depend on systematic integration of imaging methodologies into therapeutic development rather than their use as ancillary research tools. Quantitative and longitudinal immune cell tracking may contribute to improved patient selection, early detection of off-target distribution, and objective assessment of cellular persistence. Continued methodological refinement and clinical validation will determine the role of nuclear imaging in the routine management of immune cell therapies.

## 7. Limitations of This Review

This article is a narrative review rather than a systematic review or meta-analysis. Study selection was qualitative and may be affected by publication bias, heterogeneity in imaging protocols, and uneven maturity across PET, SPECT, reporter gene, nanomaterial, and optical conversion platforms. Because many cited immune-cell imaging studies remain preclinical or early translational, direct comparison of sensitivity, cell viability, radiation dose, longitudinal performance, and clinical utility is limited. The reference expansion in this revision improves breadth, but it does not substitute for a formal systematic search strategy, risk-of-bias assessment, or quantitative pooled analysis. Accordingly, the conclusions should be interpreted as a structured translational synthesis intended to guide platform selection and future study design rather than as evidence of definitive clinical superiority of one approach over another.

## 8. Conclusions

Radionuclide-based molecular imaging provides a quantitative and clinically relevant framework for monitoring immune cell therapy in translational oncology. Direct radiolabeling remains useful for early biodistribution and safety assessment, whereas NIS reporter gene imaging enables repeated, viability-dependent evaluation of cell persistence. Radionuclide-integrated nanoplatforms can improve intracellular signal retention and may support theranostic design, while Cerenkov-based methods provide complementary preclinical validation. Together, these approaches may help optimize cell dose, identify off-target distribution, evaluate persistence, and guide safer integration of immune cell therapy into oncology practice. Future clinical translation will depend on standardized PET/SPECT protocols, radiopharmaceutical nomenclature, GMP-compatible manufacturing, ethical gene-transfer strategies, and prospective validation in well-defined patient populations.

## Figures and Tables

**Table 1:** Translational Foundations Supporting Nuclear Imaging for Immune Cell Tracking.

Category	Key References	Core Contribution	Relevance to Immune Cell Tracking
NIS discovery	[1,2,3]	Cloning, transport mechanism, stoichiometry	Molecular basis of tracer uptake
Physiological & clinical characterization	[4,5,6,7,8,9]	Regulation, Endocrine application	Safety and regulatory familiarity
NIS substrate spectrum	[25,69,70,71,72,73]	PET/SPECT-compatible tracers including [^18^F]TFB, [^124^I]NaI, [^123^I]NaI, and [^99^ᵐTc]TcO_4_^−^	Supports modality selection for longitudinal tracking
Extrathyroidal expression	[10,11]	Expression in non-thyroid tissues	Adaptability to engineered cells
Reporter gene concept	[12,13,14]	Radioisotope concentrator gene therapy	Feasibility of ectopic expression
PET reporter validation	[15,16,17,18,67,68]	Quantitative PET imaging of hNIS	Longitudinal in vivo monitoring
[^18^F]TFB development	[69]	Introduction of PET-compatible NIS tracer	Improved imaging resolution
Human dosimetry & safety	[70,71]	Biodistribution and radiation assessment	Clinical translation
Clinical diagnostic validation	[25,72]	Tumor detection and comparison to iodine imaging	Proof of clinical utility

**Table 2:** Positron emission tomography (PET) and single-photon emission computed tomography (SPECT) radionuclide-based immune cell tracking platforms and representative references.

Strategy	PET Platform(s)	SPECT Platform(s)	Time Window/ Longitudinal Capability	Main Translational Use	Key Refs.
Direct radiolabeling	[^89^Zr]Zr-oxine; [^64^Cu]Cu-based labels	[^111^In]In-oxine; [^99^ᵐTc]Tc-HMPAO	Short-term; limited by decay, efflux, cell division	Early biodistribution and safety assessment	[19,20,21,22,23,24,43,44,45,46,47,48,49,50,59,60,61,62,63,64]
NIS reporter gene	hNIS + [^124^I]NaI; hNIS + [^18^F]TFB	hNIS + [^123^I]NaI; hNIS + [^99^ᵐTc]TcO_4_^−^; hNIS + [^131^I]NaI	Repeated imaging; viability-dependent signal	Longitudinal tracking of viable engineered cells	[1,2,3,4,5,6,7,8,9,10,11,12,13,14,15,16,17,18,25,26,27,28,67,68,69,70,71,72,73,74,75,76,77,78,79,80,81,82,83]
Oncolytic/viral NIS platforms	NIS vectors + [^124^I]NaI/[^18^F]TFB	NIS vectors + [^123^I]NaI/[^131^I]NaI/[^99^ᵐTc]TcO_4_^−^	Repeated imaging depending on expression stability	Gene-delivery and radiovirotherapy monitoring	[29,30,31,32,33,34,81,82,83]
Radionuclide-integrated nanomaterials	[^64^Cu]Cu-, [^89^Zr]Zr-, radioiodine-AuNPs	[^99^ᵐTc]Tc-, [^198^Au]Au-, radioiodine-AuNPs	Moderate; depends on retention and cell fate	Signal retention and theranostic design	[35,36,37,38,39,40,41,42,65,66]
Cerenkov luminescence imaging	β^+^ PET radionuclide-based CLI	Not primary; comparator γ-tracers when applicable	Isotope-dependent; mainly preclinical	Tracer validation and PET/SPECT-optical cross-validation	[51,52,53,54,55,56,57,58]

**Table 3:** Functional stratification of radionuclide-based immune cell imaging platforms.

Platform	Signal Origin and Biological Readout	Strengths	Limitations	Optimal Translational Use	Refs.
Direct radiolabeling	Preloaded intracellular radionuclide; early biodistribution independent of viability	Simple workflow; quantitative early trafficking; clinically feasible GMP labeling	Physical decay, efflux, proliferation dilution, no viability specificity	Immediate post-infusion pharmacokinetics and safety mapping	[19,20,21,22,23,24,43,44,45,46,47,48,49,50,59,60,61,62,63,64]
NIS reporter gene imaging	Transporter-mediated tracer uptake; viable-cell persistence	Repeated imaging; signal linked to living engineered cells; PET and SPECT tracer options	Requires gene transfer; physiologic uptake in thyroid, salivary glands, stomach; regulatory complexity	Long-term persistence monitoring in gene and cell therapy	[1,2,3,4,5,6,7,8,9,10,11,12,13,14,15,16,17,18,25,26,27,28,67,68,69,70,71,72,73,74,75,76,77,78,79,80,81,82,83]
Radionuclide-integrated nanomaterials	Structurally retained isotope; intracellular signal retention	No genetic modification; improved radionuclide retention; theranostic design	Long-term biocompatibility, degradation, and clearance require further evaluation	Preclinical and early translational immune-cell tracking	[35,36,37,38,39,40,41,42,65,66]
Cerenkov luminescence imaging	Optical photons generated by radioactive decay	Sensitive small-animal imaging; supports PET-optical validation	Limited tissue penetration; not suitable as stand-alone deep human imaging	Preclinical validation and radiochemistry development	[51,52,53,54,55,56,57,58]

## Data Availability

No new data were created or analyzed in this study.

## Abbreviations

The following abbreviations are used in this manuscript:

AJR	American Journal of Roentgenology
AuNPs	Gold Nanoparticles
BRAF	B-Raf Proto-Oncogene
CAR	Chimeric Antigen Receptor
CLI	Cerenkov Luminescence Imaging
CT	Computed Tomography
DC	Dendritic Cell
EJNMMI	European Journal of Nuclear Medicine and Molecular Imaging
GMP	Good Manufacturing Practice
hNIS	Human Sodium Iodide Symporter
I-124	Iodine-124
I-131	Iodine-131
KHIDI	Korea Health Industry Development Institute
NK	Natural Killer
NIS	Sodium Iodide Symporter
NRF	National Research Foundation
PET	Positron Emission Tomography
PET/CT	Positron Emission Tomography/Computed Tomography
RIe-AuNPs	Radionuclide-Embedded Gold Nanoparticles
SPECT	Single-Photon Emission Computed Tomography
TFB	Tetrafluoroborate
RPT	Radiopharmaceutical therapy
[^18^F]F	Fluorine-18 radionuclide prefix used for fluorine-18-labeled radiopharmaceuticals
[^64^Cu]Cu	Copper-64
[^89^Zr]Zr	Zirconium-89
[^89^Zr]Zr-oxine	Zirconium-89 Oxinate Complex
[^99^ᵐTc]Tc	Technetium-99m
[^198^Au]Au	Gold-198

## References

[1] Dai G., Levy O., Carrasco N. (1996). Cloning and characterization of the thyroid iodide transporter. Nature.

[2] Smanik P.A., Liu Q., Furminger T.L., Ryu K., Xing S., Mazzaferri E.L., Jhiang S.M. (1996). Cloning of the human sodium iodide symporter. Biochem. Biophys. Res. Commun..

[3] Riesco-Eizaguirre G., Santisteban P., De la Vieja A. (2021). The complex regulation of NIS expression and activity in thyroid and extrathyroidal tissues. Endocr. Relat. Cancer.

[4] Spitzweg C., Nelson P.J., Wagner E., Bartenstein P., A Weber W., Schwaiger M., Morris J.C. (2021). The sodium iodide symporter (NIS): Novel applications for radionuclide imaging and treatment. Endocr. Relat. Cancer.

[5] Arczewska K.D., Godlewska M., Krasuska W., Lyczkowska A., Kiedrowski M., Czarnocka B. (2022). Expression of pendrin and NIS iodide transporters in human breast tumor and peri-tumoral tissue. Arch. Med. Sci..

[6] Fu M., Gao Y., Guo W., Meng Q., Jin Q., Yang R., Yang Y., Zhang Y., Zhang W. (2022). Mechanisms of Sodium/Iodide Symporter-Mediated Mammary Gland Iodine Compensation during Lactation. Nutrients.

[7] Gadisi R.P., Naicker M., Naidoo S. (2025). The extra-thyroidal distribution of sodium iodide symporter. Front. Endocrinol..

[8] Lee J.H., Jung K.H., Mina K., Lee K.-H. (2022). Extracellular vesicles deliver sodium iodide symporter protein and promote cancer cell radioiodine therapy. Sci. Rep..

[9] Riesco-Eizaguirre G., Santisteban P. (2006). A perspective view of sodium iodide symporter research and its clinical implications. Eur. J. Endocrinol..

[10] Demyashkin G., Guzik A., Parshenkov M., Belokopytov D., Shchekin V., Batov M., Shegai P., Kaprin A. (2025). Elevated NIS Expression Correlates with Chemoresistance in Triple-Negative Breast Cancer: Potential Link to FOXA1 Activity. Med. Sci..

[11] Demyashkin G., Kogan E., Demura T., Guzik A., Belokopytov D., Batov M., Shchekin V., Bicherova I., Shegai P., Kaprin A. (2025). Parity and NIS Expression in Atypical Cells of Triple-Negative Breast Cancer, and Prognosis. Int. J. Mol. Sci..

[12] Kitzberger C., Spellerberg R., Han Y., Schmohl K.A., Stauss C., Zach C., Kälin R.E., Multhoff G., Eiber M., Schilling F. (2023). Mesenchymal Stem Cell-mediated Image-guided Sodium Iodide Symporter (NIS) Gene Therapy Improves Survival of Glioblastoma-bearing Mice. Clin. Cancer Res..

[13] Spellerberg R., Benli-Hoppe T., Kitzberger C., Hageneier M., Schwenk N., Öztürk Ö., Steiger K., Multhoff G., Eiber M., Schilling F. (2022). Dual EGFR- and TfR-targeted gene transfer for sodium iodide symporter gene therapy of glioblastoma. Mol. Ther. Oncolytics.

[14] Darabi N., Keshavarz M., Nabipour I., Assadi M. (2023). The sodium iodide symporter (NIS) as theranostic gene in preclinical therapy of extra-thyroidal malignancies. Clin. Transl. Imaging.

[15] Acton P.D., Zhou R. (2005). Imaging reporter genes for cell tracking with PET and SPECT. Q. J. Nucl. Med. Mol. Imaging.

[16] Kemler I., Karamched B., Neuhauser C., Dingli D. (2021). Quantitative imaging and dynamics of tumor therapy with viruses. FEBS J..

[17] Backhaus P., Pentlow K.S., Ho A.L., Mauguen A., A Fagin J., Pillarsetty N.V.K., Lyashchenko S.K., Burnazi E., Ghossein R.A., Chhabra S. (2024). [18F]TFB PET/CT misses intense [124I]iodine-avid metastases after redifferentiation therapy in metastatic thyroid cancer. EJNMMI Res..

[18] Khoshnevisan A., Jauregui-Osoro M., Shaw K., Baguña Torres J., Young J.D., Ramakrishnan N.K., Jackson A., Smith G.E., Gee A.D., Blower P.J. (2016). [18F]tetrafluoroborate as a PET tracer for the sodium/iodide symporter: The importance of specific activity. EJNMMI Res..

[19] Kiraga L., Kucharzewska P., Paisey S.J., Cheda L., Domanska A., Rogulski Z., Rygiel T.P., Boffi A., Krol M. (2021). Nuclear imaging for immune cell tracking in vivo: Comparison of various cell labeling methods and their application. Coord. Chem. Rev..

[20] Galli F., Varani M., Lauri C., Silveri G.G., Onofrio L., Signore A. (2021). Immune cell labelling and tracking: Implications for adoptive cell transfer therapies. EJNMMI Radiopharm. Chem..

[21] Lechermann L.M., Lau D., Attili B., Aloj L., Gallagher F.A. (2021). In vivo cell tracking using PET: Opportunities and challenges for clinical translation in oncology. Cancers.

[22] Ashmore-Harris C., Iafrate M., Saleem A., Fruhwirth G.O. (2020). Non-invasive reporter gene imaging of cell therapies, including T cells and stem cells. Mol. Ther..

[23] Roca M., de Vries E.F.J., Jamar F., Israel O., Signore A. (2010). Guidelines for the labelling of leucocytes with 111In-oxine. Eur. J. Nucl. Med. Mol. Imaging.

[24] de Vries E.F.J., Roca M., Jamar F., Israel O., Signore A. (2010). Guidelines for the labelling of leucocytes with 99mTc-HMPAO. Eur. J. Nucl. Med. Mol. Imaging.

[25] Diocou S., Volpe A., Jauregui-Osoro M., Boudjemeline M., Chuamsaamarkkee K., Man F., Blower P.J., Ng T., Mullen G.E.D., Fruhwirth G.O. (2017). [18F]tetrafluoroborate-PET/CT enables sensitive tumor and metastasis in vivo imaging in a sodium iodide symporter-expressing tumor model. Sci. Rep..

[26] Chaurasiya S., Yang A., Zhang Z., Lu J., Valencia H., Kim S.-I., Woo Y., Warner S.G., Olafsen T., Zhao Y. (2022). Comprehensive preclinical study supporting clinical trial of oncolytic chimeric poxvirus CF33-hNIS-anti-PDL1 to treat breast cancer. Mol. Ther. Methods Clin. Dev..

[27] Han Y., Koehler V.F., Nagarajah J., Schmohl K.A., Stauss C., Schwenk N., Spellerberg R., Kitzberger C., Kumbrink J., Zach C. (2025). TGF-β1-based restoration of sodium iodide symporter expression in radioiodine-refractory differentiated thyroid cancer via engineered MSCs. Mol. Ther..

[28] Riesco-Eizaguirre G., Rodríguez I., De la Vieja A., Costamagna E., Carrasco N., Nistal M., Santisteban P. (2009). The BRAFV600E oncogene induces sodium iodide symporter repression. Cancer Res..

[29] Shah S., Lucke-Wold B. (2024). Image-guided mesenchymal stem cell NIS radionuclide therapy for glioblastoma. Cancers.

[30] Zhang Z., Yang A., Chaurasiya S., Park A.K., Lu J., Kim S.-I., Warner S.G., Yuan Y.-C., Liu Z., Han H. (2022). CF33-hNIS-antiPDL1 virus primes pancreatic ductal adenocarcinoma for enhanced therapy. Cancer Gene Ther..

[31] Chaurasiya S., Kim S.I., O’Leary M., Park A.K., Lu J., Kang S., Zhang Z., Yang A., Woo Y., Fong Y. (2021). Toward comprehensive imaging of oncolytic viroimmunotherapy. Mol. Ther. Oncolytics.

[32] Rand J., Yamauchi D., Chaurasiya S., Zhang J., Deshpande S., Chong L., Seiz A., Meisen H., Fong Y., Yuan Y. (2025). hNIS-based imaging to monitor treatment with the novel oncolytic virus CF33-hNIS-antiPDL1 in humans with advanced triple negative breast cancer. Front. Oncol..

[33] Dingli D., Diaz R.M., Bergert E.R., O’Connor M.K., Morris J.C., Russell S.J. (2003). Genetically targeted radiotherapy for multiple myeloma. Blood.

[34] Msaouel P., Iankov I.D., Allen C., Aderca I., Federspiel M.J., Tindall D.J., Morris J.C., Koutsilieris M., Russell S.J., Galanis E. (2009). Noninvasive imaging and radiovirotherapy of prostate cancer using an oncolytic measles virus expressing the sodium iodide symporter. Mol. Ther..

[35] Goel M., Mackeyev Y., Krishnan S. (2023). Radiolabeled nanomaterials for cancer diagnostics and therapy: Recent advances. Cancer Nanotechnol..

[36] Zhen Z., Feng L., Liu H., Chen M., Chen J., Wang J. (2025). Radionuclide-labeled nanomaterials for tumor therapy: Recent progress and perspectives. Mater. Today Bio.

[37] Sun N., Wang T., Zhang S. (2024). Radionuclide-labelled nanoparticles for cancer combination therapy and imaging. J. Nanobiotechnol..

[38] Wang R., Liu H., Antal B., Wolterbeek H.T., Denkova A.G. (2024). Ultrasmall gold nanoparticles radiolabeled with iodine-125 for targeted cancer therapy. ACS Appl. Bio Mater..

[39] Lee S.B., Lee S.W., Jeong S.Y., Yoon G., Cho S.J., Kim S.K., Lee I.K., Ahn B.C., Lee J., Jeon Y.H. (2017). Engineering of radioiodine-labeled gold core-shell nanoparticles for dendritic cell trafficking imaging. ACS Appl. Mater. Interfaces.

[40] Lee S.B., Lee Y.J., Cho S.J., Kim S.K., Lee S.W., Lee J., Lim D.K., Jeon Y.H. (2018). Antigen-free radionuclide-embedded gold nanoparticles for dendritic cell maturation and tracking. Adv. Healthc. Mater..

[41] Lee S.B., Lee J.-E., Cho S.J., Chin J., Kim S.K., Lee I.K., Lee S.W., Lee J., Jeon Y.H. (2019). Crushed gold shell nanoparticles labeled with radioactive iodine as a theranostic nanoplatform. Nano Micro Lett..

[42] Shen W., Zhou H., Liu T., Pei P., Huang J., Yi X., Yang K. (2020). The potential clinical applications of radionuclide labeled/doped gold-based nanomaterials. Radiat. Med. Prot..

[43] Young D.J., Edwards A.J., Quiroz Caceda K.G., Liberzon E., Barrientos J., Hong S.G., Turner J., Choyke P.L., Arlauckas S.P., Lazorchak A.S. (2025). In vivo tracking of ex-vivo-generated 89Zr-oxine-labeled plasma cells by PET in a non-human primate model. Mol. Ther..

[44] Khan A., Man F., Muhammad Faruqu F., Kim J., Al-Salemee F., Minino A., Volpe A., Liam-Or R., Simpson P., Fruhwirth G. (2022). PET Imaging of Small Extracellular Vesicles via [89Zr]Zr(oxinate)4 Direct Radiolabeling. Bioconjug. Chem..

[45] Bystrom J., Da Costa M.P., Carrascal-Miniño A., Qureshi A., Keeling G.P., Pham T.T., Sunassee K., Carroll E.C., Garrod-Ketchley C., Schroth J. (2025). Impact of age on the homing potential of 89Zr-radiolabelled CD8+ T cells (pilot preclinical PET tracking study). Sci. Rep..

[46] Teng M., Liang X., Liu H., Li Z., Gao X., Zhang C., Cheng H., Chen H., Liu G. (2024). Cerenkov radiation shining a light for cancer theranostics. Nano Today.

[47] Xu P., Lin S., Wang Y., Abdukayum A., Wang Y. (2024). Radionuclide-based Cerenkov luminescence in biomedicine: Current research progress and future perspectives. TrAC. Trends Anal. Chem..

[48] Liu N., Su X., Sun X. (2022). Cerenkov radiation-activated probes for deep cancer theranostics: A review. Theranostics.

[49] Pham T.T., Chenoweth A., Patel N., Banu A., Osborn G., Blower P.J., Karagiannis S.N., Ma M.T. (2024). In vivo PET imaging of 89Zr-labeled NK and CAR-NK cells. J. Nucl. Med..

[50] Massicano A.V.F., Bartels J.L., Jeffers C.D., Crenshaw B.K., Houson H., Mueller C., Younger J.W., Knapp P., McConathy J.E., Lapi S.E. (2021). Production of [89Zr]oxinate4 and cell radiolabeling for human use. J. Label. Comp. Radiopharm..

[51] Pratt E.C., Skubal M., Mc Larney B., Causa-Andrieu P., Das S., Sawan P., Araji A., Riedl C., Vyas K., Tuch D. (2022). Prospective testing of clinical Cerenkov luminescence imaging against standard-of-care nuclear imaging for tumour location. Nat. Biomed. Eng..

[52] Ruggiero A., Holland J.P., Lewis J.S., Grimm J. (2010). Cerenkov luminescence imaging of medical isotopes. J. Nucl. Med..

[53] Thorek D.L.J., Ogirala A., Beattie B.J., Grimm J. (2013). Quantitative imaging of disease signatures through radioactive decay signal conversion. Nat. Med..

[54] Alborghetti L., Vurro F., Belloli S., Rainone P., Valtorta S., Capialbi M.M., Lacavalla M.A., Pizzardi S., Moresco R.M., Boschi F. (2025). Cerenkov luminescence microscopy: A novel approach for high-resolution radiotracer imaging. iScience.

[55] Yang Z., Pang T.T., Wu Z.J., Yan T.Y., Yu J.M., Wang X.Y., Liu D., Lu X.J., Kang X.Y., Li G.Y. (2025). Construction of a high-sensitivity Cherenkov luminescence imaging system in tumor models. EJNMMI Res..

[56] Spyratou E., Kokkinogoulis K., Tsigaridas G., Kareliotis G., Platoni K., Makropoulou M., Efstathopoulos E.P. (2023). Novel Biophotonic Techniques for Phototherapy Enhancement: Cerenkov Radiation as a Bridge between Ionizing and Non-Ionizing Radiation Treatment. J. Nanother..

[57] Thorek D.L.J., Das S., Grimm J. (2014). Molecular imaging using nanoparticle quenchers of Cerenkov luminescence. Small.

[58] van Dam R.M., Chatziioannou A.F. (2021). Cerenkov luminescence imaging in radiochemistry development. Front. Phys..

[59] Man F., Khan A.A., Carrascal-Miniño A., Blower P.J., de Rosales R.T.M. (2020). A kit formulation for the preparation of [89Zr]Zr(oxinate)4 for PET cell tracking: White blood cell labelling and comparison with [111In]In(oxinate)3. Nucl. Med. Biol..

[60] Weist M.R., Starr R., Aguilar B., Chea J., Miles J.K., Poku E., Gerdts E., Yang X., Priceman S.J., Forman S.J. (2018). PET of adoptively transferred chimeric antigen receptor T cells with 89Zr-oxine. J. Nucl. Med..

[61] Man F., Lim L., Volpe A., Gabizon A., Shmeeda H., Draper B., Parente-Pereira A.C., Maher J., Blower P.J., Fruhwirth G.O. (2019). In vivo PET tracking of 89Zr-labeled Vγ9Vδ2 T cells to mouse xenograft breast tumors activated with liposomal alendronate. Mol. Ther..

[62] Sta Maria N.S., Barnes S.R., Weist M.R., Khawli L.A., Pachipulusu V., Lin S.W., Zheng L., Cohrs D., Liu X., Hu P. (2021). Spatio-temporal biodistribution of 89Zr-oxine-labeled CAR T cells in mice. Sci. Rep..

[63] Kurebayashi Y., Choyke P.L., Sato N. (2021). Imaging of cell-based therapy using 89Zr-oxine ex vivo cell labeling for positron emission tomography. Nanotheranostics.

[64] Koyasu S., Minor H.A., Asiedu K.O., Choyke P.L., Sato N. (2025). Zirconium-89-oxine cell tracking by PET reveals in vivo trafficking of monocyte-macrophage lineage cells. Pharmaceuticals.

[65] Lee S.B., Ahn S.B., Lee S.W., Jeong S.Y., Ghilsuk Y., Ahn B.-C., Kim E.-M., Jeong H.-J., Lee J., Lim D.-K. (2016). Radionuclide-embedded gold nanoparticles for enhanced dendritic cell-based cancer immunotherapy, sensitive and quantitative tracking of dendritic cells with PET and Cerenkov luminescence. NPG Asia Mater..

[66] Daems N., Michiels C., Lucas S., Baatout S., Aerts A. (2021). Gold nanoparticles meet medical radionuclides. Nucl. Med. Biol..

[67] Dittmann M., Gonzalez Carvalho J.M., Rahbar K., Schäfers M., Claesener M., Riemann B., Seifert R. (2020). Incremental diagnostic value of [18F]tetrafluoroborate PET-CT compared to [131I]iodine scintigraphy in recurrent differentiated thyroid cancer. Eur. J. Nucl. Med. Mol. Imaging.

[68] Groot-Wassink T., Aboagye E.O., Wang Y., Lemoine N.R., Reader A.J., Vassaux G. (2004). Quantitative imaging of Na/I symporter transgene expression using positron emission tomography in the living animal. Mol. Ther..

[69] Niu M., Qin J., Wang L., Hu H., Shi J., Yang X., Zhang Y., Zhang J. (2022). Evaluation of [18F]tetrafluoroborate as a Potential PET Imaging Agent in a Sodium Iodide Symporter-Transfected Cell Line A549 and Endogenous NIS-Expressing Cell Lines MKN45 and K1. Mol. Imaging.

[70] O’Doherty J.W., Jauregui-Osoro M., Brothwood T., Szyszko T.A., Marsden P., O’Doherty M.J., Cook G.J.R., Blower P.J., Lewington V.J. (2017). [18F]tetrafluoroborate ([18F]TFB), a PET probe for imaging sodium/iodide symporter expression: Whole-body biodistribution, safety and radiation dosimetry in thyroid cancer patients. J. Nucl. Med..

[71] Piccardo A., Fiz F., Righi S., Raffa S., Riondato M., Bottoni G., Bauckneht M., Massollo M., Rizzo A., Naseri M.S.Z. (2025). [18F]Tetrafluoroborate, a new NIS PET/CT radiopharmaceutical: An overview focused on differentiated thyroid cancer. Eur. Thyroid. J..

[72] Ventura D., Dittmann M., Büther F., Schäfers M., Rahbar K., Hescheler D., Claesener M., Schindler P., Riemann B., Seifert R. (2024). Diagnostic performance of [18F]TFB PET/CT compared with therapeutic activity [131I]iodine SPECT/CT and [18F]FDG PET/CT in recurrent differentiated thyroid carcinoma. J. Nucl. Med..

[73] Coenen H.H., Gee A.D., Adam M., Antoni G., Cutler C.S., Fujibayashi Y., Jeong J.M., Mach R.H., Mindt T.L., Pike V.W. (2017). Consensus nomenclature rules for radiopharmaceutical chemistry: Setting the record straight. Nucl. Med. Biol..

[74] Penheiter A.R., Russell S.J., Carlson S.K. (2012). The sodium iodide symporter (NIS) as an imaging reporter for gene, viral, and cell-based therapies. Curr. Gene Ther..

[75] Ahn B.C. (2012). Sodium iodide symporter for nuclear molecular imaging and gene therapy: From bedside to bench and back. Theranostics.

[76] Brader P., Serganova I., Blasberg R.G. (2013). Noninvasive molecular imaging using reporter genes. J. Nucl. Med..

[77] Jauregui-Osoro M., Sunassee K., Weeks A.J., Berry D.J., Paul R.L., Cleij M., Banga J.P., O’doherty M.J., Marsden P.K., Clarke S.E.M. (2010). Synthesis and biological evaluation of [18F]tetrafluoroborate: A PET imaging agent for thyroid disease and reporter gene imaging of the sodium/iodide symporter. Eur. J. Nucl. Med. Mol. Imaging.

[78] Jiang H., Schmit N.R., Koenen A.R., Bansal A., Pandey M.K., Glynn R.B., Kemp B.J., Delaney K.L., Dispenzieri A., Bakkum-Gamez J.N. (2017). Safety, pharmacokinetics, metabolism and radiation dosimetry of [18F]tetrafluoroborate ([18F]TFB) in healthy human subjects. EJNMMI Res..

[79] Shalaby N., Xia Y., Kelly J.J., Sanchez-Pupo R., Martinez F., Fox M.S., Thiessen J.D., Hicks J.W., Scholl T.J., Ronald J.A. (2024). Imaging CAR-NK cells targeted to HER2 ovarian cancer with human sodium-iodide symporter-based positron emission tomography. Eur. J. Nucl. Med. Mol. Imaging.

[80] Jacob J., Nadkarni S., Volpe A., Peng Q., Tung S.L., Hannen R.F., Mohseni Y.R., Scotta C., Marelli-Berg F.M., Lechler R.I. (2021). Spatiotemporal in vivo tracking of polyclonal human regulatory T cells reveals a role for innate immune cells in Treg transplant recruitment. Mol. Ther. Methods Clin. Dev..

[81] Vandergaast R., Khongwichit S., Jiang H., DeGrado T.R., Peng K.-W., Smith D.R., Russell S.J., Suksanpaisan L. (2020). Enhanced noninvasive imaging of oncology models using the NIS reporter gene and bioluminescence imaging. Cancer Gene Ther..

[82] Concilio S.C., Russell S.J., Peng K.W. (2021). A brief review of reporter gene imaging in oncolytic virotherapy and gene therapy. Mol. Ther. Oncolytics.

[83] Volpe A., Pillarsetty N.V.K., Lewis J.S., Ponomarev V. (2021). Applications of nuclear-based imaging in gene and cell therapy: Probe considerations. Mol. Ther. Oncolytics.

[84] Dohan O., De la Vieja A., Paroder V., Riedel C., Artani M., Reed M., Ginter C.S., Carrasco N. (2003). The sodium/iodide symporter (NIS): Characterization, regulation, and medical significance. Endocr. Rev..

[85] Micali S., Bulotta S., Puppin C., Territo A., Navarra M., Bianchi G., Damante G., Filetti S., Russo D. (2014). Sodium iodide symporter (NIS) in extrathyroidal malignancies: Focus on breast and urological cancer. BMC Cancer.

[86] Kogai T., Taki K., Brent G.A. (2006). Enhancement of sodium/iodide symporter expression in thyroid and lactating breast. Endocr. Relat. Cancer.

[87] Ryan J., Curran C.E., Hennessy E., Newell J., Morris J.C., Kerin M.J., Dwyer R.M. (2011). The sodium iodide symporter (NIS) and potential regulators in normal, benign, and malignant human breast tissue. PLoS ONE.

[88] US Food and Drug Administration (2020). Long Term Follow-Up After Administration of Human Gene Therapy Products: Guidance for Industry.

[89] US Food and Drug Administration (2024). Considerations for the Development of Chimeric Antigen Receptor (CAR) T Cell Products: Guidance for Industry.

[90] Eisenman D., Swindle S. (2022). Food and Drug Administration guidance on design of clinical trials for gene therapy products with potential for genome integration or genome editing and associated long-term follow-up of research subjects. Appl. Biosaf..

[91] Doctor A., Seifert V., Ullrich M., Hauser S., Pietzsch J. (2020). Three-dimensional cell culture systems in radiopharmaceutical cancer research. Cancers.

[92] Abdollahi H., Saboury B., Soltani M., Shi K., Uribe C., Rahmim A. (2023). Radiopharmaceutical therapy on-a-chip: A perspective on microfluidic-driven digital twins towards personalized cancer therapies. Sci. Bull..

[93] Nayak P., Bentivoglio V., Varani M., Signore A. (2023). Three-dimensional in vitro tumor spheroid models for evaluation of anticancer therapy: Recent updates. Cancers.

[94] Petreus T., Cadogan E., Hughes G., Smith A., Reddy V.P., Lau A., O’cOnnor M.J., Critchlow S., Ashford M., O’cOnnor L.O. (2021). Tumour-on-chip microfluidic platform for assessment of drug pharmacokinetics and treatment response. Commun. Biol..

[95] Fang L., Liu Y., Qiu J., Wan W. (2022). Bioprinting and its use in tumor-on-a-chip technology for anticancer drug screening. Int. J. Bioprint..

[96] Liu X., Fang J., Huang S., Wu X., Xie X., Wang J., Liu F., Zhang M., Peng Z., Hu N. (2021). Tumor-on-a-chip: From bioinspired design to biomedical application. Microsyst. Nanoeng..

[97] Regmi S., Fu A., Luo K.Q. (2022). Applications of microfluidics and organ-on-a-chip in cancer research. Biosensors.

